# Impaired humoral immunity following COVID-19 vaccination in HTLV-1 carriers

**DOI:** 10.1186/s12879-024-09001-z

**Published:** 2024-01-17

**Authors:** Takuro Kameda, Atae Utsunomiya, Nobuaki Otsuka, Yoko Kubuki, Taisuke Uchida, Kotaro Shide, Ayako Kamiunten, Nobuaki Nakano, Masahito Tokunaga, Takayoshi Miyazono, Yoshikiyo Ito, Kentaro Yonekura, Toshiro Kawakita, Keiichi Akizuki, Yuki Tahira, Masayoshi Karasawa, Tomonori Hidaka, Ayaka Konagata, Norifumi Taniguchi, Yuma Nagatomo, Fumiko Kogo, Koichiro Shimizu, Hiroaki Ueno, Junzo Ishizaki, Naoya Takahashi, Yoshihiko Ikei, Michihiro Hidaka, Hideki Yamaguchi, Kazuya Shimoda

**Affiliations:** 1 Division of Hematology, Diabetes, and Endocrinology, Department of Internal Medicine, Faculty of Medicine, University of Miyazaki, 5200 Kihara, Kiyotake, Miyazaki 889-1692 Japan; 2 Department of Hematology, Imamura General Hospital, Kagoshima, Japan; 3 Youkikai Ikei Hospital, Kobayashi, Japan; 4 Department of Dermatology, Imamura General Hospital, Kagoshima, Japan; 5 National Hospital Organization Kumamoto Medical Center, Kumamoto, Japan; 6 Department of Internal Medicine, Aisenkai Nichinan Hospital, Nichinan, Japan

**Keywords:** COVID-19, HTLV-1, SARS-COV-2 spike protein, Humoral immunity, Vaccination

## Abstract

**Background:**

Whether human T-lymphotropic virus type 1 (HTLV-1) carriers can develop sufficient humoral immunity after coronavirus disease 2019 (COVID-19) vaccination is unknown.

**Methods:**

To investigate humoral immunity after COVID-19 vaccination in HTLV-1 carriers, a multicenter, prospective observational cohort study was conducted at five institutions in southwestern Japan, an endemic area for HTLV-1. HTLV-1 carriers and HTLV-1-negative controls were enrolled for this study from January to December 2022. During this period, the third dose of the COVID-19 vaccine was actively administered. HTLV-1 carriers were enrolled during outpatient visits, while HTLV-1-negative controls included health care workers and patients treated by participating institutions for diabetes, hypertension, or dyslipidemia. The main outcome was the effect of HTLV-1 infection on the plasma anti-COVID-19 spike IgG (IgG-S) titers after the third dose, assessed by multivariate linear regression with other clinical factors.

**Results:**

We analyzed 181 cases (90 HTLV-1 carriers, 91 HTLV-1-negative controls) after receiving the third dose. HTLV-1 carriers were older (median age 67.0 vs. 45.0 years, *p* < 0.001) and more frequently had diabetes, hypertension, or dyslipidemia than did HTLV-1-negative controls (60.0% vs. 27.5%, *p* < 0.001). After the third dose, the IgG-S titers decreased over time in both carriers and controls. Multivariate linear regression in the entire cohort showed that time since the third dose, age, and HTLV-1 infection negatively influenced IgG-S titers. After adjusting for confounders such as age, or presence of diabetes, hypertension, or dyslipidemia between carriers and controls using the overlap weighting propensity score method, and performing weighted regression analysis in the entire cohort, both time since the third dose and HTLV-1 infection negatively influenced IgG-S titers.

**Conclusions:**

The humoral immunity after the third vaccination dose is impaired in HTLV-1 carriers; thus, customized vaccination schedules may be necessary for them.

**Supplementary Information:**

The online version contains supplementary material available at 10.1186/s12879-024-09001-z.

## Background

People with cancer have significantly increased morbidity and mortality from coronavirus disease 2019 (COVID-19), compared with the general public [1, 2]. This is most apparent in patients with hematological malignancies, with a risk of severe course and/or death of 27–36% [3, 4]. In addition, although most of the general population and patients with cancer acquire anti-COVID-19 spike protein IgG (IgG-S) antibodies after receiving mRNA- or adenovirus-based COVID-19 vaccines, patients with hematological malignancies, particularly those receiving anti-CD20 immunotherapy, do not [5–7].

Human T-lymphotropic virus type 1 (HTLV-1) is a retrovirus that causes adult T-cell leukemia/lymphoma (ATL) and progressive nervous system disorders known as HTLV-1-associated myelopathy or tropical spastic paraparesis (HAM/TSP). Individuals infected with HTLV-1 on their infantile days through breast milk from HTLV-1-carrier mothers, or those infected by sexual contact with semen containing HTLV-1-infected leukocytes, become HTLV-1 carriers; the total number of HTLV-1 carriers worldwide is estimated to be between 5 and 10 million [8]. A small percentage of HTLV-1 carriers develop ATL or HAM/TSP, and most HTLV-1 carriers do not develop any HTLV-1-related disease during their lifetime. However, even in the absence of HTLV-1-related diseases, HTLV-1 carriers receive some degree of immunomodulation from HTLV-1, affecting their susceptibility to infection by several pathogens [9], and this effect may extend to COVID-19. Therefore, we evaluated IgG-S antibody titers in HTLV-1 carriers who received the third (booster) dose of the COVID-19 vaccine.

## Methods

### Study design and population

A multicenter prospective cohort study was conducted at five institutions within the Miyazaki/Kagoshima/Kumamoto Prefecture, an HTLV-1 endemic area in southwestern Japan, to investigate the humoral immunity to COVID-19 vaccines in HTLV-1 carriers. From January to December 2022, HTLV-1 carriers and HTLV-1-negative controls, who had received the third dose of the COVID-19 vaccine, were recruited for this study. HTLV-1-negative controls included volunteers working as health care workers at participating institutions and patients treated at participating institutions for diabetes, hypertension, or dyslipidemia (Supplementary Fig. 1). Those with active ATL or HAM/TSP and those with other malignancies or autoimmune diseases undergoing treatment were excluded from the study. Blood samples (plasma) were collected prospectively during routine hospital visits or when they were enrolled as volunteers and were tested for antibody titers. IgG-S titers were tested to assess each participant’s humoral immunity to the COVID-19 vaccine. To exclude the effect of previous severe acute respiratory syndrome coronavirus 2 (SARS-CoV-2) infection on IgG-S titers, a medical interview and measurement of anti-nucleocapsid IgG (IgG-N) antibody titers were performed, and those with COVID-19 infection history or with IgG-N positivity were excluded from the study. As IgG-S antibody titers peaked approximately 2 weeks after the third dose, followed by a gradual decrease over time [10, 11], samples collected less than 14 days after vaccination were excluded from the analyses. Clinical data were collected using a case report form and included age at enrollment; sex; body mass index; comorbidities, including diabetes, hypertension, dyslipidemia, malignant tumors, and autoimmune diseases; medications being administered; past medical history and treatment, such as malignant tumors; drinking habits; smoking habits. Regarding COVID-19 vaccination history, the date of each vaccination and the type of vaccine the participant had received, BNT162b2 (Pfizer) or mRNA-1273 (Moderna), were recorded. Drinking habits were surveyed based on the number of drinking days per week. Smoking habits were surveyed based on the following three levels: “never had a habit before,” “had a habit in the past,” and “still have a habit.” This study was conducted following the Declaration of Helsinki and was approved by the Institutional Ethics Committee of the Faculty of Medicine, University of Miyazaki, and other participating institutes (O-1061). Written informed consent was obtained from all study participants.

### SARS-CoV-2 antibody analyses

For plasma samples collected, IgG-S and IgG-N antibody titers were measured using Lumipulse® G SARS-CoV-2 S-IgG, SARS-CoV-2 N-IgG, and the Lumipulse® G1200 assay system (FUJIREBIO Inc., Tokyo, Japan), or using Elecsys® Anti-SARS-CoV-2 S RUO, Elecsys® Anti-SARS-CoV-2 RUO, and Cobas® 8000 e801 module (Roche Diagnostics, Rotkreuz, Switzerland), both according to the manufacturer’s instructions. Both Lumipulse® G and Cobas® 8000 are assay systems for quantitatively measuring IgG-type antibodies in specimens based on chemiluminescent enzyme immunoassay (CLEIA) technology, using a specific two-step immunoassay method. Measurements of IgG-S (arbitrary units per milliliter (AU)/mL in the Lumipulse system and U/mL in the Cobas module) were converted to WHO International Binding Antibody Units (BAU/mL), using conversion factors provided by the reagent companies, and were used for plotting and regression analysis [12]. The WHO defines cutoff values for anti-SARS-CoV-2-S1-receptor binding domain IgG of approximately 44–53 BAU/mL, 200–300 BAU/mL, and 700–800 BAU/mL as low, mid, and high titers, respectively; a recent study also supported 50 BAU/mL as the cutoff between negative and positive samples [13, 14]. For IgG-N antibody titers, we used 1.0 AU/mL for SARS-CoV-2 N-IgG or index value 1.0 for Elecsys® Anti-SARS-CoV-2 RUO as the cutoff between negative and positive samples, according to the manufacturer’s instructions.

### Statistical analysis

Patient characteristics were compared between groups using the Fisher’s exact and Mann–Whitney U tests. In addition, IgG-S antibody titers were compared between the groups using the Mann–Whitney U test. To identify the factors affecting IgG-S titers, univariate and multivariate linear regression analyses were performed. For all regression analyses, the response variable was defined as log10-transformed IgG-S titers. For multivariate linear regression analysis of IgG-S titers (log10-transformed), explanatory variables included HTLV-1 infection, proviral load of HTLV-1, and clinical factors reported to be associated with IgG-S titers in healthy individuals or healthcare workers: age, sex, BMI, drinking and smoking habits, presence of diabetes, hypertension, or dyslipidemia, COVID-19 vaccination history involving different types of combinations, and the time lag between vaccine dose and sample collection [15–22]. Furthermore, the propensity score method using overlap weights was employed to adjust for confounding variables [23–25]. Overlap weighting assigns weights to each patient based on the probability of that patient belonging to the opposite group. Specifically, HTLV-1 carriers are weighted by the probability of being HTLV-1-negative controls (1 − PS), and HTLV-1-negative controls are weighted by the probability of being HTLV-1 carriers (PS), where PS represents the propensity score. After adjusting for confounders between HTLV-1 carriers and HTLV-1-negative controls, weighted linear regression analysis and weighted Mann–Whitney U tests for IgG-S titers were performed. Results were considered significant at *p* < 0.05. Statistical analyses were performed using the R (version 4.1.2) and its packages ggplot2, tableone, PSweight, and gt-summary.

## Results

Overall, 112 HTLV-1 carriers and 100 HTLV-1-negative controls comprising health care workers (*n* = 82) and patients with diabetes, hypertension, or dyslipidemia (*n* = 18), were enrolled in this study. Participants or samples were excluded according to the exclusion criteria, and HTLV-1 carriers did not include patients with HAM and ATL (Supplementary Fig. 1). Finally, 90 HTLV-1 carriers and 91 HTLV-1-negative controls (health care workers, *n* = 76; patients with either diabetes, hypertension, or dyslipidemia, *n* = 15), who received the third dose of the COVID-19 vaccine, were included in the analysis. The median ages at vaccination were 67.0 years and 45.0 years for HTLV-1 carriers and HTLV-1-negative controls, respectively (*p* < 0.001) (Table 1). As 10 of the health care workers in the control group had diabetes, hypertension, or dyslipidemia, totally, the control group included 25 patients (27.5%) with diabetes, hypertension, or dyslipidemia. Compared with HTLV-1 negative controls, HTLV-1 carriers were more likely to have a higher BMI and to have either diabetes, hypertension, or dyslipidemia. In addition, HTLV-1 carriers were more likely to have been vaccinated with different combinations of the BNT162b2 and mRNA-1273 COVID-19 vaccine. This different distribution was primarily because the BNT162b2 vaccine was preferentially given to health care workers who volunteered for the study. The median HTLV-1 proviral load in HTLV-1 carriers was 20.6 [5.7, 48.4] copies/1000 PBMCs (median [interquartile range]).

**Table 1:** Background of participants whose samples were collected after the third vaccine dose

Variable	Overall	HTLV-1-negative control	HTLV-1 carrier	p
n	181	91	90	
Age (median [IQR])	58.0 [44.0, 69.0]	45.0 [33.0, 54.0]	67.0 [59.0, 71.0]	< 0.001
Sex, female/male, n (%)	125/56 (69.1/30.9)	65/26 (71.4/28.6)	60/30 (66.7/33.3)	0.523
Higher BMI, n (%)	13 (7.2)	3 (3.3)	10 (11.1)	0.048
Drinking habit, n (%)	51 (28.2)	26 (28.6)	25 (27.8)	1
Smoking habit, n (%)	20 (11.0)	9 (9.9)	11 (12.2)	0.644
Diabetes, n (%)	18 (9.9)	8 (8.8)	10 (11.1)	0.629
Hypertension, n (%)	59 (32.6)	18 (19.8)	41 (45.6)	< 0.001
Dyslipidemia, n (%)	39 (21.5)	11 (12.1)	28 (31.1)	0.002
Presence of diabetes, hypertension, or dyslipidemia, n (%)	79 (43.6)	25 (27.5)	54 (60.0)	< 0.001
Treatment history of malignancy, n (%)	5 (2.8)	2 (2.2)	3 (3.3)	0.682
Time lag between vaccination and sampling, months (median [IQR])	2.20 [1.33, 3.93]	1.57 [1.33, 3.65]	2.97 [1.71, 4.33]	0.012
COVID-19 vaccination history involving different types of combinations	50 (27.6)	13 (14.3)	37 (41.1)	< 0.001
COVID-19 vaccination combinations				< 0.001
3 doses of BNT162b2	127 (70.2)	74 (81.3)	53 (58.9)	
2 doses of BNT162b2 and 1 dose of mRNA-1273	48 (26.5)	13 (14.3)	35 (38.9)	
1 dose of BNT162b2 and 2 doses of mRNA-1273	2 (1.1)	0 (0.0)	2 (2.2)	
3 doses of mRNA-1273	4 (2.2)	4 (4.4)	0 (0.0)	

Abbreviations: HTLV-1, human T-lymphotropic virus type 1; BMI, Body Mass Index; p, p value between the HTLV-1 carrier and HTLV-1-negative control groups. Higher BMI is defined as “BMI > = 30.” Drinking habit is defined as “drinking alcohol more than 3 days per week.” Smoking habit is defined as “still have a habit.”

**Fig. 1:**
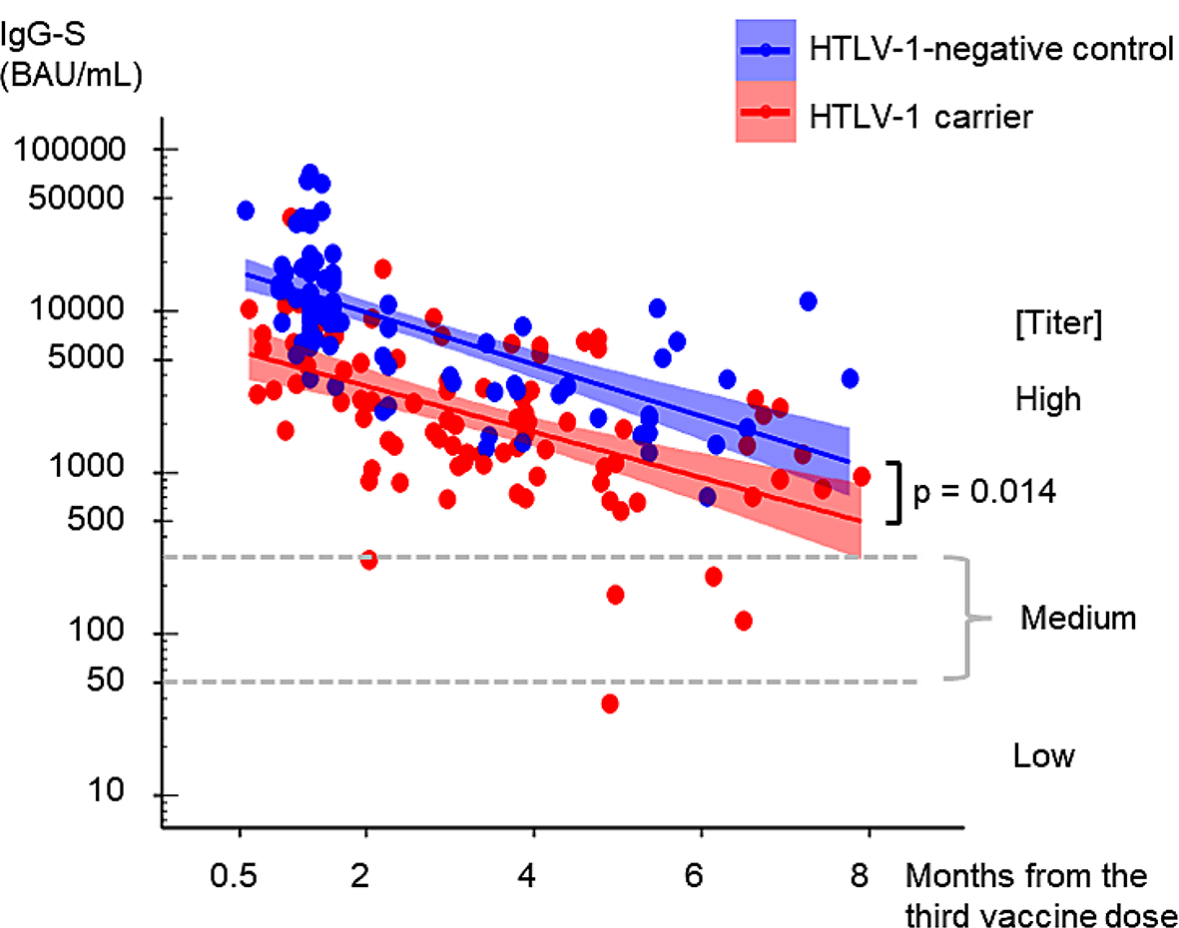
Dynamics of anti-COVID-19 spike protein IgG (IgG-S) antibody titers after the third vaccine dose. IgG-S antibody titers in HTLV-1 carriers and HTLV-1-negative controls along the time from the third vaccine dose. The x-axis represents the sampling time point based on the date of the third dose (months). The y-axis represents IgG-S antibody titers (BAU/mL) on a log10 scale. Univariate regression lines for IgG-S titers by the time from the third dose to sample collection are shown with 95% CIs. The dashed lines indicate the cutoff values that distinguish low, medium, and high titers of anti-SARS-CoV-2-S1-receptor binding domain IgG as defined by the WHO. HTLV-1, human T-lymphotropic virus type 1; BAU, binding antibody units; IgG-S, anti-COVID-19 spike protein IgG; CI, confidence interval; WHO, World Health Organization

Except for one HTLV-1 carrier with 37.2 BAU/mL at 5 months after the third vaccine dose, all HTLV-1 carriers and HTLV-1-negative controls were positive for IgG-S antibodies (50 BAU/mL) after the third dose (Fig. 1). This exceptional elderly 76-year-old HTLV-1 carrier with a proviral load of 6.1 copies/1000 PBMCs had no other reported factors associated with impaired humoral immunity to the anti-COVID-19 vaccine such as heavy smoking or drinking habits. Univariate linear regression of IgG-S titers with a time lag after the third dose, showed that the time lag negatively influenced IgG-S titers both in HTLV-1 carriers and the control group (the coefficient of a time lag in HTLV-1-negative controls, β = -0.371, *p* < 0.001; and that in HTLV-1 carriers, β = -0.328, *p* < 0.001). We performed multivariate linear regressions for both HTLV-1 carriers and HTLV-1-negative controls, analyzing IgG-S titers in relation to time lag, HTLV-1 infection, and other clinical factors reported to be associated with IgG-S titers after the second dose in healthy individuals or healthcare workers [15–22]; these include age, BMI, diabetes, hypertension, dyslipidemia, and diverse COVID-19 vaccination histories (Table 1). We observed that time lag inversely impacted IgG-S titers in both groups, while age showed a similar effect exclusively in HTLV-1 carriers (Table 2).

**Table 2:** Factors affecting IgG-S titers by multivariate linear regression for HTLV-1 carriers and HTLV-1-negative controls

	HTLV-1-negative control				HTLV-1 carrier		
Variable	Estimate	SE	p		Estimate	SE	p
(Intercept)	10.38	0.301	0.000		11.62	0.734	0.000
Time lag, month	-0.369	0.049	0.000		-0.358	0.056	0.000
Age	-0.008	0.007	0.256		-0.038	0.010	0.000
Male gender	-0.169	0.216	0.435		-0.082	0.281	0.772
Higher BMI	0.255	0.455	0.578		-0.211	0.336	0.532
Drinking habit	-0.244	0.189	0.200		-0.012	0.300	0.967
Smoking habit	0.083	0.281	0.769		-0.521	0.329	0.117
Presence of diabetes, hypertension, or dyslipidemia	0.151	0.207	0.467		-0.085	0.217	0.696
COVID-19 vaccination history involving different types of combinations	-0.275	0.265	0.302		-0.173	0.205	0.402

Abbreviations: BMI, Body Mass Index; SE, standard error; p, p value. Higher BMI is defined as “BMI > = 30.” Drinking habit is defined as “drinking alcohol more than 3 days per week.” Smoking habit is defined as “still have a habit.”

**Fig. 2:**
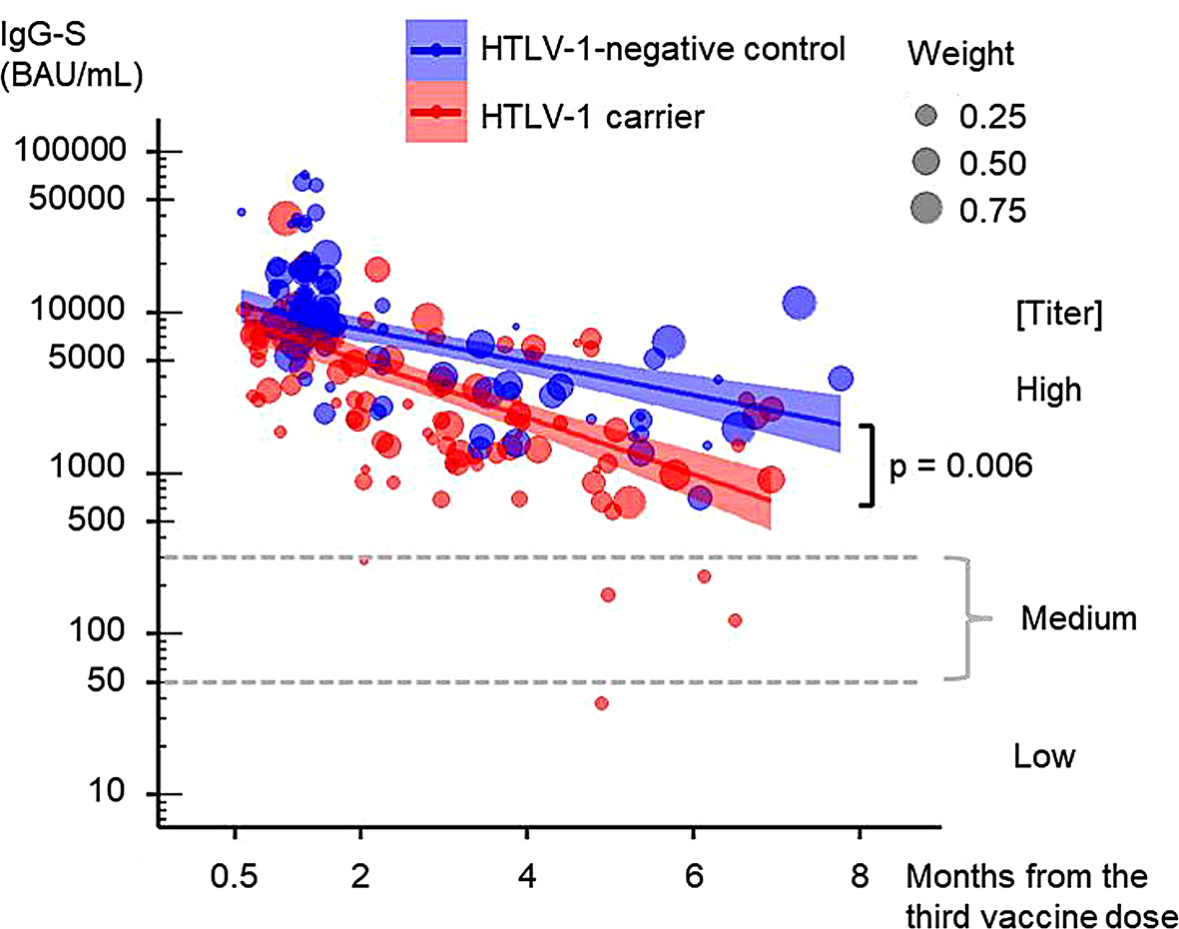
Dynamics of anti-COVID-19 spike protein IgG (IgG-S) antibody titers after the third vaccine dose for adjusted samples. IgG-S antibody titers in HTLV-1 carriers and HTLV-1-negative controls along the time from the third vaccine dose for adjusted samples. The x-axis represents the sampling time point based on the date of the third dose (months). The y-axis represents IgG-S antibody titers (BAU/mL) on a log10 scale. The size of each dot represents the weight of each sample, calculated by propensity score method using overlap weights. Weighted univariate regression lines for IgG-S titers by the time from the third dose to sample collection are shown with 95% CIs. The dashed lines indicate the cutoff values that distinguish low, medium, and high titers of anti-SARS-CoV-2-S1-receptor binding domain IgG as defined by the WHO. HTLV-1, human T-lymphotropic virus type 1; BAU, binding antibody units; IgG-S, anti-COVID-19 spike protein IgG; CI, confidence interval; WHO, World Health Organization

Furthermore, when conducting multivariate linear regression in the entire cohort, time lag, age, and HTLV-1-infection negatively affected IgG-S titers (Table 3). To refine the understanding of HTLV-1 infection’s impact on IgG-S titers, particularly considering age and the presence of diabetes, hypertension, or dyslipidemia, we adjusted for background differences between HTLV-1 carriers and HTLV-1-negative controls. This adjustment was achieved using the propensity score method with overlap weights (Supplementary Table 1, Supplementary Fig. 2) [23–25]. Post-adjustment, the multivariate linear regressions of the entire cohort revealed that both time lag and HTLV-1 infection continued to adversely affect IgG-S titers (Table 3; Fig. 2).

**Table 3:** Factors affecting IgG-S titers by multivariate linear regression for the entire cohort with or without adjustment

	Without adjustment				With adjustment		
Variable	Estimate	SE	p		Estimate	SE	p
(Intercept)	10.85	0.272	< 0.001		10.32	0.711	< 0.001
Time lag, month	-0.356	0.036	< 0.001		-0.304	0.058	< 0.001
HTLV-1 infection	-0.465	0.188	0.014		-0.501	0.179	0.006
Age	-0.020	0.006	0.001		-0.009	0.012	0.450
Male gender	-0.081	0.158	0.610		-0.213	0.200	0.288
Higher BMI	-0.131	0.257	0.610		-0.053	0.252	0.833
Drinking habit	-0.066	0.159	0.679		0.136	0.142	0.338
Smoking habit	-0.182	0.211	0.392		-0.157	0.161	0.332
Presence of diabetes, hypertension, or dyslipidemia	0.061	0.147	0.678		-0.051	0.146	0.728
COVID-19 vaccination history involving different types of combinations	-0.161	0.155	0.300		-0.311	0.161	0.056

The propensity score method using overlap weights was employed to adjust for confounding

Abbreviations: HTLV-1, human T-lymphotropic virus type 1; BMI, Body Mass Index; SE, standard error; p, p value. Higher BMI is defined as “BMI > = 30.” Drinking habit is defined as “drinking alcohol more than 3 days per week.” Smoking habit is defined as “still have a habit”

## Discussion

In this study, we demonstrated that HTLV-1 carriers had lower IgG-S antibody titers than did HTLV-1-negative controls after a third dose of the COVID-19 vaccine, suggesting an impaired humoral immunity following COVID-19 vaccination.

The antibody titers acquired after the second vaccine dose in patients with hematological malignancy were significantly lower than those in healthy controls, particularly in those with lymphoid malignancies undergoing immunotherapy and/or chemotherapy [5]. The third dose may boost humoral immunity in patients with hematological malignancies. Among 25 patients with positive IgG-S titers before the third dose, 23 (92%) had increased IgG-S titers after the third dose [6]. However, patients who initially tested negative for antibodies (seronegative) still tested negative even after the third dose. Of the 23 patients with a history of anti-CD20 treatment, those treated within 12 months before the third dose responded poorly, compared with those receiving the same drug at least 12 months before the third dose. In another report, most participants who were treatment-naïve or had completed systemic treatment more than 24 weeks before the third dose had improved antibody levels; however, 29% of the participants still had lower IgG-S levels after the third dose [7]. Decreased antibody titers acquired after a vaccine dose were also reported in people living with human immunodeficiency virus (PLWH) and patients receiving hemodialysis. After the second and third doses of COVID-19 mRNA-based vaccine, IgG-S titers were lower in PLWH compared with healthy controls [26]. This trend was accentuated in the subgroup of patients with lower CD4^+^ T-cell counts [26]. In hemodialysis patients, only 24% and 77% of patients had more than 500 BAU/mL 6 months after the second and third doses of COVID-19 vaccination, respectively [27].

As with patients with hematological malignancies with anti-CD20 treatment or PLWH, HTLV-1 carriers had impaired humoral immunity after the third vaccine dose. A previous study demonstrated a clear correlation between IgG-S and neutralizing antibodies after vaccination in patients with hematological malignancies [6]. Therefore, HTLV-1 carriers with a third vaccine dose might not develop a humoral immune protective effect against COVID-19 to a similar extent as HTLV-1-negative controls do. Higher levels of IgG-S were sustained beyond 4 months after the third dose in HTLV-1-negative controls, which was consistent with a report where the third dose sustained high levels of neutralizing antibodies against SARS-CoV-2, at 6 months following vaccination in healthy individuals [28]. Furthermore, another report comparing antibody waning after the second and third doses showed that the waning of IgG-S levels was slower after the third dose than after the second dose [29]. In the adjusted regression analysis for the entire cohort in our study, time since the third dose and HTLV-1 infection still negatively influenced IgG-S titers. Age, smoking, drinking, higher BMI, presence of diabetes, hypertension, or dyslipidemia have harmed IgG-S titers after the second dose in HTLV-1-negative populations [15–21]. However, the effects of these unfavorable factors were attenuated after the third dose [30]. Similarly, these factors did not affect IgG-S titers after the third dose in HTLV-1-negative controls in our study. However, age still negatively influenced IgG-S titers in HTLV-1 carriers along with time lag, suggesting a distinct immunological background in each group.

HTLV-1 infection is associated with altered expression of immunosuppressive or antigen-presenting molecules such as programmed death receptor-1 (PD-1), programmed cell death ligand 1 (PD-L1), or human leukocyte antigen (HLA) class II on CD4^+^ T cells [31–34]. Notably, this aberrant expression is not confined to HTLV-1-infected cells; it also extends to non-infected antigen-presenting cells within the microenvironment, potentially leading to a diminished humoral response following vaccination [33, 34]. As PD-1 expression on CD4^+^ T cells in healthy aged individuals has been reported to correlate with the decreased expansion and maintenance of spike-specific CD4^+^ T cells and CD8^+^ T cells following anti-COVID-19 vaccination [35], HTLV-1 infection may contribute to the decreased humoral immunity against COVID-19 vaccination. Indeed, humoral and CD4^+^ T-cell responses to tetanus toxoid were impaired in HTLV-1 carriers, partly because of a decrease in the intensity of HLA–DR isotype expression on monocytes and the low frequency of dendritic cell subsets, possibly resulting in impaired antigen presentation to T-cells [36]. HTLV-1-specific immunomodulation might contribute to the impaired humoral immunity following COVID-19 vaccination.

Our study report on the humoral immunity following the administration of mRNA-based anti-COVID-19 vaccine to HTLV-1 carriers. Additionally, Esfehani et al. recently reported that HTLV-1 carriers who received a second or third dose of protein-based Sinopharm’s anti-COVID-19 vaccine showed impaired humoral immunity 28 days after vaccination, compared with HTLV-1 negative controls [37]. Whether protein-based or mRNA-based, humoral immunity appears to be impaired in HTLV-1 carriers after administration of anti-COVID vaccine. These observations may be useful in determining the time of the following vaccine dose in HTLV-1 carriers.

This study has some limitations. There were some background differences between HTLV-1 carriers and HTLV-1-negative controls. In addition, timing of blood collection is not constant, which depends on each participant’s routine clinical visits. Despite these limitations, this study provides basic data on this neglected infectious disease, which is an important public health issue in some endemic regions.

## Conclusion

Humoral immunity to COVID-19 vaccines is impaired in HTLV-1 carriers. The protective effect of humoral immunity in HTLV-1 carriers may last only for a shorter period than that in HTLV-1-negative controls. Our observations may help to understand the susceptibility of HTLV-1 carriers to COVID-19 and develop an optimal vaccination schedule.

### Electronic supplementary material

Below is the link to the electronic supplementary material.

**Supplementary Material 1: File format:** Word (.DOC). **Supplementary Table 1:** Patient characteristics adjusted by propensity score method using overlap weights. **Supplementary Figure 1:** Patient flow in this study. **Supplementary Figure 2:** Assessment of absolute standardized mean differences in covariates and the distribution of weighted cases using the overlap weighting method

## Data Availability

The datasets used and/or analyzed during the current study are available from the corresponding author on reasonable request.

## Abbreviations

COVID-19: coronavirus disease 2019
HTLV-1: human T-lymphotropic virus type 1
ATL: Adult T-cell Leukemia
HAM/TSP: HTLV-1-associated myelopathy or tropical spastic paraparesis
SARS-CoV-2: Severe Acute Respiratory Syndrome Coronavirus 2
AU: Arbitrary Units per milliliter
BAU: Binding Antibody Units
