# Supplementary material for: Impaired humoral immunity following COVID-19 vaccination in HTLV-1 carriers
Source: BMC Infect Dis. 2024 Jan 17;24:96. doi: 10.1186/s12879-024-09001-z (PMC10792913; doi:10.1186/s12879-024-09001-z)
Supplement: Supplementary file 1 — Supplementary Material 1: File format: Word (.DOC). Supplementary Table 1: Patient characteristics adjusted by propensity score method using overlap weights. Supplementary Figure 1: Patient flow in this study. Supplementary Figure 2: Assessment of absolute standardized mean differences in covariates and the distribution of weighted cases using the overlap weighting method [file 12879_2024_9001_MOESM1_ESM.docx]

**Additional File 1**

**Supplementary Table 1** lists patient characteristics adjusted by propensity score method using overlap weights.

**Supplementary Figure 1** shows patient flow in this study.

**Supplementary Figure 2** shows the assessment of absolute standardized mean differences in covariates and the distribution of weighted cases using the overlap weighting method.

| **Supplementary Table 1. Patient Characteristics Adjusted by Propensity Score Method Using Overlap Weights.** | | | |
| --- | --- | --- | --- |
| **Variable** | **Control** | **Carrier** | **SMD** |
| Age (median [IQR]) | 57.9 (10.2) | 57.9 (9.2) | <0.001 |
| Sex, female/male, n (%) | 8.6 (40.1) | 8.6 (40.1) | <0.001 |
| Higher BMI, n (%) | 1.1 (4.9) | 1.1 (4.9) | <0.001 |
| Drinking habit, n (%) | 7.6 (35.2) | 7.6 (35.2) | <0.001 |
| Smoking habit, n (%) | 2.4 (11.4) | 2.4 (11.4) | <0.001 |
| Diabetes, n (%) | 2.7 (12.5) | 1.9 (8.9) | 0.117 |
| Hypertension, n (%) | 8.9 (41.3) | 7.6 (35.3) | 0.124 |
| Dyslipidemia, n (%) | 4.6 (21.4) | 6.5 (30.3) | 0.204 |
| Presence of diabetes, hypertension, or dyslipidemia, n (%) | 10.4 (48.3) | 10.4 (48.3) | <0.001 |
| Treatment history of malignancy, n (%) | 1.9 (9.8) | 3.1 (16.0) | 0.188 |
| COVID-19 vaccination history involving different types of combinations | 5.9 (27.5) | 5.9 (27.5) | <0.001 |
| Abbreviations: BMI, Body Mass Index; PBMCs, Peripheral Blood Mononuclear Cells; SMD: standardized mean difference. Higher BMI is defined as “BMI ≥ 30.” Drinking habit is defined as "drinking alcohol more than 3 days per week." Smoking habit is defined as "still have a habit." | | | |

**
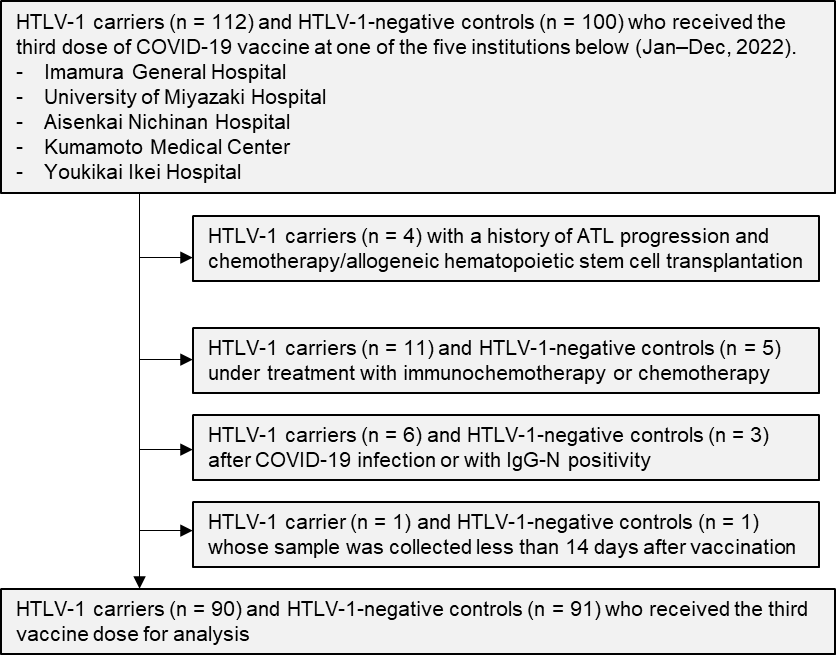
**

**Supplementary Figure 1. Patient flow in this study.**

Overall, 112 HTLV-1 carriers and 100 HTLV-1-negative controls were enrolled in this study. The following participants were excluded from the analysis: participants with a history of ATL progression and chemotherapy/allogeneic hematopoietic stem cell transplantation; those under treatment with immunochemotherapy or chemotherapy, participants after COVID-19 infection or with IgG-N positivity; and participants whose samples were collected less than 14 days after vaccination. Finally, 90 HTLV-1 carriers and 91 HTLV-1-negative controls who received a third dose of the COVID-19 vaccine were prepared for analysis. HTLV-1, human T-lymphotropic virus type 1; COVID-19, coronavirus disease 2019; ATL, adult T-cell leukemia/lymphoma

**
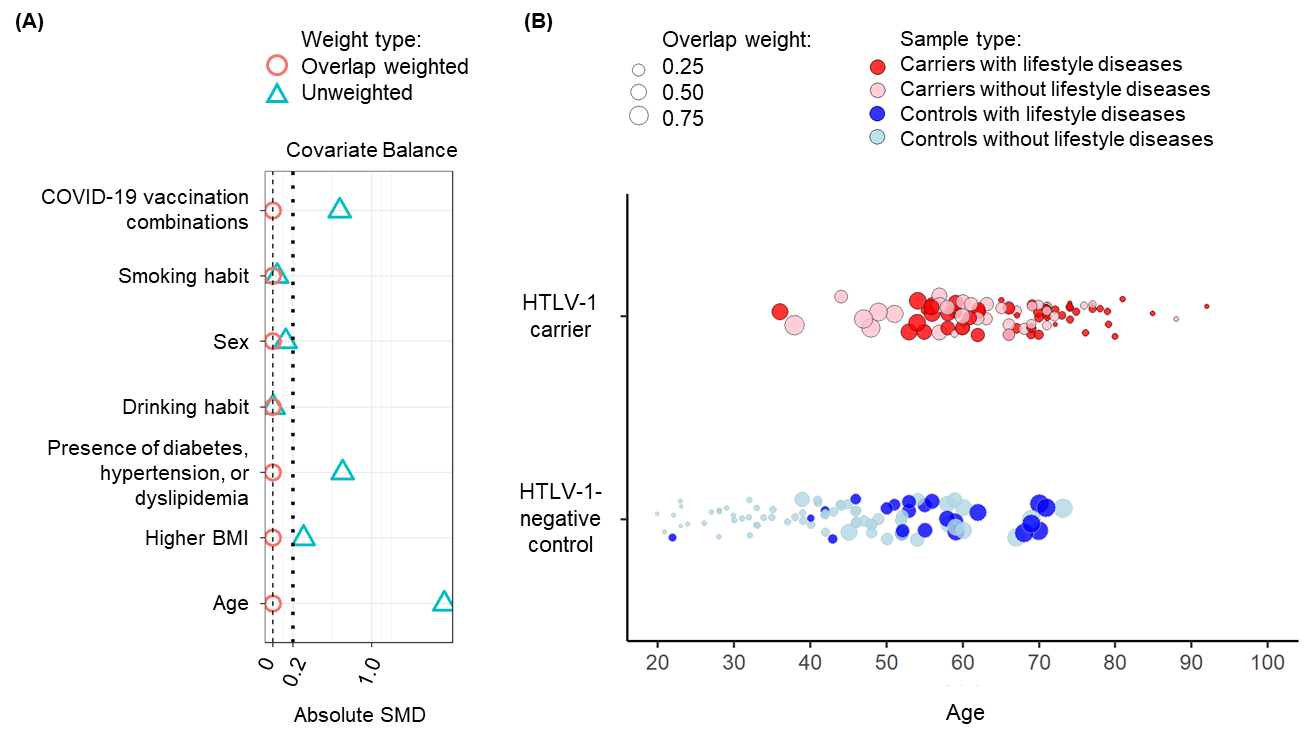
**

**Supplementary Figure 2.**

**(A) Assessment of absolute standardized mean differences in covariates between unweighted and weighted samples.**

Absolute standardized mean differences in covariates were assessed between unweighted and weighted samples of HTLV-1 carriers and HTLV-1-negative controls. Confounding factors were adjusted by propensity score method using overlap weights, resulting in significantly lower standardized mean differences. SMD, standardized mean difference; BMI, Body Mass Index.

**(B) Distribution of cases weighted by the overlap weights.**

The x-axis represents age, the y-axis represents HTLV-1 infection status, dot size corresponds to the overlap weight, and color denotes sample type based on HTLV-1 infection and presence of lifestyle-related diseases. A similar weight is assigned to the population aged 50–70 years with approximately 50% prevalence of lifestyle-related diseases among both HTLV-1 carriers and HTLV-1-negative controls.
